# A B-ARR-mediated cytokinin transcriptional network directs hormone cross-regulation and shoot development

**DOI:** 10.1038/s41467-018-03921-6

**Published:** 2018-04-23

**Authors:** Mingtang Xie, Hongyu Chen, Ling Huang, Ryan C. O’Neil, Maxim N. Shokhirev, Joseph R. Ecker

**Affiliations:** 1 Plant Biology Laboratory, and Genomic Analysis Laboratory, The Salk Institute for Biological Studies, La Jolla, CA 92037 USA; 2 Howard Hughes Medical Institute, The Salk Institute for Biological Studies, La Jolla, CA 92037 USA; 3 Department of Computer Science, Dartmouth College, Hanover, NH 03755 USA; 4 The Razavi Newman Integrative Genomics and Bioinformatics Core Facility, The Salk Institute for Biological Studies, La Jolla, CA 92037 USA; 5 Bioinformatics Program, University of California at San Diego, La Jolla, CA 92093 USA

**Keywords:** Shoot apical meristem, Cytokinin

## Abstract

Cytokinin fulfills its diverse roles in planta through a series of transcriptional responses. We identify the in vivo DNA binding site profiles for three genetically redundant type-B ARABIDOPSIS RESPONSE REGULATORS (B-ARRs): ARR1, ARR10, and ARR12. The expression and genome-wide DNA binding locations of the three *B-ARRs* extensively overlap. Constructing a primary cytokinin response transcriptional network reveals a recurring theme of widespread cross-regulation between the components of the cytokinin pathway and other plant hormone pathways. The B-ARRs are found to have similar DNA binding motifs, though sequences flanking the core motif were degenerate. Cytokinin treatments amalgamate the three different B-ARRs motifs to identical DNA binding signatures (AGATHY, H(a/t/c), Y(t/c)) which suggests cytokinin may regulate binding activity of B-ARR family members. Furthermore, we find that *WUSCHEL*, a key gene required for apical meristem maintenance, is a cytokinin-dependent B-ARR target gene, demonstrating the importance of the cytokinin transcription factor network in shoot development.

## Abstract (web summary)

Cytokinin regulates gene expression by activation of ARR transcription factors. Here, the authors use ChIP-seq to show how three type B-ARRs mediate cytokinin response in *Arabidopsis* and provide evidence that cytokinin regulates meristem development by promoting B-ARR binding to *WUSCHEL*.

## Introduction

Cytokinin, an *N*^*6*^-substituted adenine derivative, along with other phytohormones orchestrates almost every aspect of plant growth and development, including meristem function, vascular development, biotic and abiotic stresses, and leaf senescence^1–5^. Cytokinin was first discovered for its ability to promote cell division over fifty years ago^6^. In the past twenty years, its own biosynthesis and signaling pathways and diverse roles in regulating cellular processes have been revealed by both forward and reverse genetic screens^2,7–12^. Cytokinin employs a two-component multi-step phosphorelay for its perception and signaling transduction^12–14^. In Arabidopsis, there are three cytokinin receptors (*ARABIDOPSIS HISTIDINE KINASEs; AHK2, 3, 4*) and eleven type-B response regulators (*ARABIDOSPIS RESPONSE REGULATORs; B-ARRs*)^8,15^. In cytokinin signaling cascades, the histidine-containing phosphor-transfer proteins (AHPs) act as phosphor-transfer intermediates for various AHK-AHP-B-ARR modules^16^. Genetic analysis also revealed that only higher order mutants of each family render pronounced developmental phenotypes, indicating redundancy in the cytokinin signaling pathway^8^.

The cytokinin transcriptional response centrally affects the family of ARRs. Type-B ARRs (B-ARRs) are transcription factors (TFs) with a GARP-like DNA binding domain at their C-termini and a receiver domain at their N-termini. Type-A ARRs (A-ARRs) are similar to the N-termini receiver domain of B-ARRs but do not possess a DNA binding domain. A-ARRs are negative cytokinin regulators but their mechanism of inhibition in cytokinin signaling remains unknown^12^. The DNA binding domain and protein nuclear localization signal domain at the C-terminus of B-ARRs are responsible for B-ARRs entering the nucleus and binding to their targets while their activation domain is responsible for the activation of cytokinin transcriptional responses. The presence of the receiver domain in B-ARRs is thought to cause inhibition at low cytokinin levels and may block the upstream phosphorelay to B-ARRs. It is postulated that the receiver domain masks the DNA binding domain of B-ARRs until its conformation is altered by cytokinin, which finally results in the activation of B-ARRs^15^. In previous genetic analyses, five Arabidopsis B-ARRs were shown to act in cytokinin signaling cascades with ARR1, ARR10, and ARR12 playing critical roles in plant growth and development^8^. The *A-ARRs* are cytokinin response genes that are the targets of B-ARR TFs^12^. However, the *B-ARRs* are not regulated at the transcriptional level by cytokinin but are post transcriptionally controlled^11^. Recently, B-ARRs were shown to be regulated at the level of protein stability, at least in part, through the ubiquitin-proteasome pathway^17^.

Previous in vitro studies have identified candidate binding motifs for the B-ARRs^18,19^ and a “golden list” of cytokinin response genes from microarray expression data and RNA-seq data^20^. However, the identity of which cytokinin responsive genes may be direct targets of the B-ARRs remains unknown. In addition, most experiments have depended upon treatment with high concentrations of cytokinin, since the targets of B-ARRs are almost impossible to identify at the endogenous levels of cytokinin in transcriptomic studies. Therefore, identification of the genome-wide targets of B-ARRs, with and without cytokinin treatment, would facilitate our understanding of the cytokinin responsive DNA regulatory elements, provide insights into cytokinin primary responsive gene expression, and potentially elucidate the mechanism(s) by which cytokinin ultimately regulates diverse physiological responses. Recently, genome-wide binding sites of ARR10 were identified by chromatin immunoprecipitation sequencing (ChIP-seq) of a tagged, over-expressed ARR10 fusion protein^21^, demonstrating the utility of in vivo DNA binding studies for cytokinin response pathway analysis.

Cytokinin plays an important but poorly understood role in the maintenance of the stem cell niche and regulation of meristem size^22,23^. First, inhibition of a subset of *A-ARRs* by *WUSCHEL* (*WUS*) has been demonstrated, although the mechanism of this repression remains unknown^24^. Since *A-ARRs* are targets of B-ARRs, it can be postulated that the repression of *A-ARRs* by *WUS* involves *B-ARRs*. Second, the *arr1/10/12* triple mutant was shown to produce a smaller size shoot apical meristem^8^. Third, genetic manipulation of cytokinin levels either by loss-of-function mutants of *LONELY GUYS*, which are involved in the one step conversion of cytokinin precursors to active cytokinin^25^, or by over-expression of cytokinin oxidase produces meristem defects^26^. Finally, plant regeneration requires the proper ratio of cytokinin and auxin^27^. Therefore, a greater understanding of the targets of B-ARRs may provide a link between the cytokinin transcriptional response and important plant developmental processes such as meristem development.

Our study aims to construct a core cytokinin transcriptional response network with a focus on systematic identification of the binding targets of the B-ARRs, the key players in cytokinin signaling. We take advantage of the power of recombineering^28^ to generate plants containing epitope-tagged *B-ARRs* and employ ChIP-seq to identify their genome-wide binding locations. Extensive targeting of multiple type-B ARRs to a common set of genes reveals a conserved core cytokinin transcriptional response network and extensive cross-regulation of the plant hormone pathways. We also demonstrate that the regulation of *WUS* by B-ARRs is critical for stem cell maintenance in the shoot apical meristem. These findings provide potential avenues to further explore the mechanism operating downstream cytokinin responses that control diverse growth and development processes.

## Results

### The protein localization of B-ARRs reveals extensive overlap

**Fig. 1:**
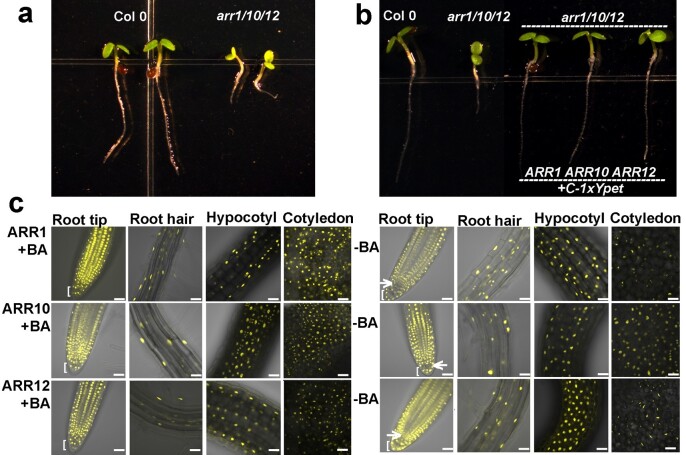
B-ARRs protein localization upon cytokinin treatment. **a** The phenotype of 3-days-old *arr1/10/12* triple mutants grown under long day (16-h light/8-h dark) conditions on MS. **b** Recombineering B-ARRs with Ypet at C-termini (+C-1xYpet) can rescue *arr1/10/12* triple mutant phenotypes. **c** The protein localization of B-ARRs in plants after 4-h treatments of 10 µM 6-BA (+BA) and DMSO (−BA). Three individual plants for each factor were imaged and representative images were presented (*n* = 3). Images of root tip regions (Root tip), root hair regions (Root hair), hypocotyls (Hypocotyl), and the adaxial side of cotyledons (Cotyledon) are shown. Arrows indicate the quiescent center. Square brackets indicate the columella cells. Scale bar = 40 µM

Previous genetic studies of the *arr1/10/12* triple mutant revealed pronounced developmental phenotypes, such as smaller size seedling and adult plants, effects likely due to a smaller shoot apical meristem and insensitivity to cytokinin treatment^2,8^. Such studies revealed that ARR1, ARR10, ARR12 are critical components of the cytokinin signaling pathway (Fig. 1a). To explore the cellular distribution pattern of these three B-ARRs, Ypet (yellow fluorescent protein)-tagged B-ARRs lines were generated using a recombineering-based gene tagging technique^28^. The advantages of this strategy are that both the expression pattern and protein location can be monitored. Moreover, because the near-by gene (cis-) regulatory information is maintained, these tagged gene constructs provide a state nearest to the native expression of the endogenous B-ARRs as is currently technically possible in plants^28^. ARR1, ARR10, and ARR12 tagged lines were generated in the Col-0 background and were used for ChIP-seq experiments. The functionality of these constructs was also confirmed by successful complementation of the *arr1/10/12* triple mutant phenotype (Fig. 1b).

Previous organ-specific expression analysis using reverse transcription PCR and promoter reporter analysis using GUS staining revealed an overlapping expression pattern of B-ARRs^8,29^. We used our ARR-recombineering lines to study the expression pattern of B-ARRs in three-days-old seedlings in the absence and presence of cytokinin (10 µM 6-Benzylaminopurine (6-BA)). We found that all three B-ARRs had similar pattern expressed in roots, hypocotyl, and cotyledons (Fig. 1c; *n* = 3 for each ARR gene). Consistent with previous findings, all three B-ARRs were localized in the nucleus^18,29–31^. However, the localization of ARR1 upon 6-BA treatment was more obvious in root tip and cotyledons than in other tissues (Fig. 1c-ARR1 + BA). In contrast, the 6-BA treatment had less impact on the localization of ARR10 compared to other tested-ARRs (Fig. 1c-ARR10). This was consistent with previous findings identifying ARR10 as the most stable B-ARR^8,32^. Additionally, the intensity of ARR12 was slightly lower than the mock treatment in the root hair region of roots (Fig. 1c-ARR12-BA). One apparent discrepancy with previous results^8,29^ was the expression of ARR1, ARR10, ARR12 in columella cells of root tips (Fig. 1c, square bracket).

### Construction of a cytokinin network using B-ARRs targets

**Fig. 2:**
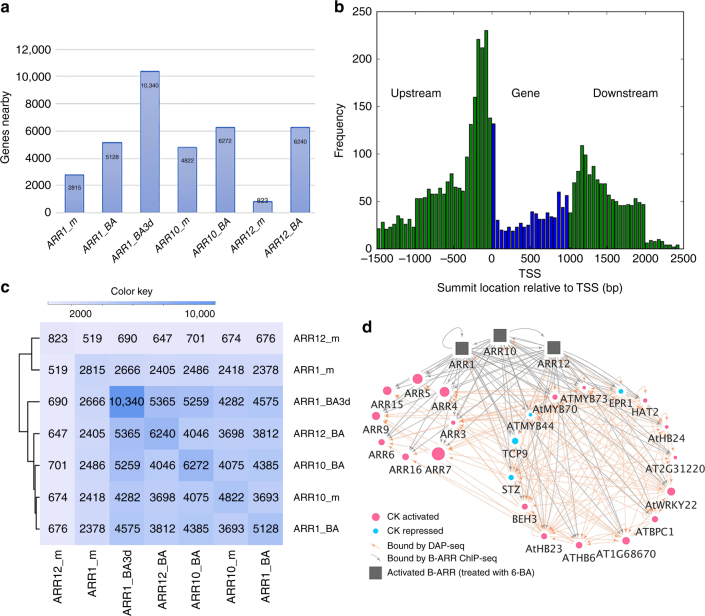
Cytokinin-dependent transcriptional response network. **a** Potential target genes near the binding sites of B-ARRs by different treatments (mock treatment (m), treatment with 10 µM 6-BA for 4 h (BA), or treatment for 3 days (BA3d)). Nearby genes were determined by B-ARR binding within 1.5 kb upstream and downstream of the gene’s annotation. The total number of nearby genes for each treatment is printed on each bar. **b** Distribution of ChIP-seq peaks around a normalized gene (1 kb) for ARR1_m. The base position relative to the transcription start site (TSS). **c** Heatmap showing number of the shared target genes of the B-ARRs TF-TF interaction network. Conditions are: mock treatment: ARR1_m, ARR10_m, ARR12_m; 4-h 10 µM 6-BA treatment: ARR1_BA, ARR12_BA; 3-day 10 µM 6-BA treatment: ARR1_BA3d. **d** A sub-network graph of the B-ARRs gene regulatory network. Nodes were either type-A ARRs or transcription factors from the core target genes of B-ARRs with DAP-seq data available. The sizes of nodes are in proportion to the max peak score of B-ARRs (Supplementary Data 5, column B). Nodes in red color are cytokinin activated while nodes in blue are cytokinin repressed. Edges in gray represent B-ARR direct bound genes and edges in salmon red represent DAP-seq bound genes. The position of nodes does not mean anything. This is the sub-network with the top 50% ranked transcription factors and the entire directed network of B-ARR regulatory network was available as Supplementary Data 6

The targets (genes near the DNA binding sites of B-ARRs) of three key B-ARRs (1, 10, 12) were identified by ChIP-seq using long-day (16-h light/8 h dark cycle) conditions at 22 °C and 3-days-old seedlings growing vertically on plates containing Murashige and Skoog (MS) medium (Fig. 2a, and Supplementary Fig. 1, Supplementary Data 1). The binding profiles of all three factors were generated either in the absence of 6-BA (endogenous level of cytokinin) or in the presence of 10 µM 6-BA (cytokinin treatment) for 4 h, or 3 days (only for ARR1). Without 6-BA treatment, 2815 (ARR1_m), 4822 (ARR10_m), and 823 (ARR12_m) targets were identified using a cutoff of *p*-value 1E-16 (MACS2 peak caller, cutoff: +/−1.5 kb of genes). Using the same standards, the cytokinin treated samples had higher numbers of targets for all three B-ARRs (5128 (ARR1_BA), 6272 (ARR10_BA), and 6240 (ARR12_BA)). An increase in the number of targets upon cytokinin treatment might result from either protein stabilization or modification by phosphorylation^11^ or both processes. Interestingly, samples treated with 10 µM 6-BA for three days showed even further increase in the number of targets for ARR1, up to approximately 10,000. B-ARR binding sites detected by ChIP-seq were highly enriched near gene transcription start sites (TSS) (Fig. 2b and Supplementary Fig. 2). They were enriched in regions 1.5 kb upstream and 1 kb downstream of genes, but enrichment dropped dramatically beyond 1 kb downstream of genes.

**Fig. 3:**
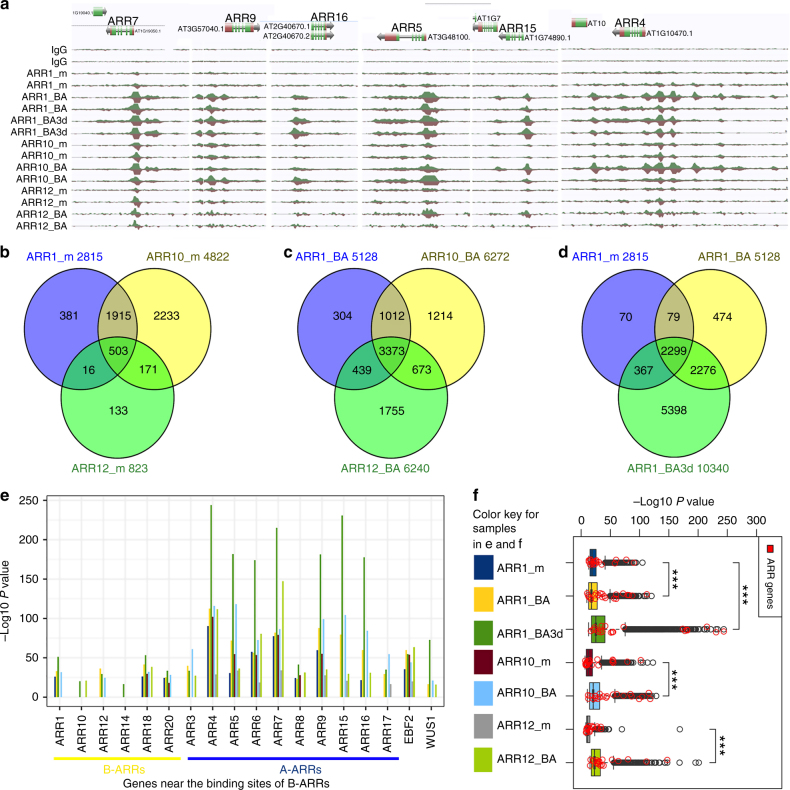
*A-ARR*s are direct targets of B-ARR transcription factors. **a** AnnoJ browser snapshots of *A-ARR* genes, targets of B-ARR TFs. **b** Venn diagram describing the number of target genes for each of three key B-ARRs in the mock treatment (m). **c** Venn diagram describing the number of target genes for each of three key B-ARRs in the 10 µM 6-BA 4-h treatment (BA). **d** Venn diagram describing the number of target genes increase for ARR1 in a cytokinin time course treatment, 0 h (m), 4 h (BA), 3 days (BA3d). **e** Peak scores (−Log10 *p-*value) of *ARRs* in various cytokinin treatments. **f** Box plot showing the distributions of the binding peak scores of B-ARR targets. *ARRs* were highlighted in red circles. Asterisks (***) indicates significant difference using Wilcoxon rank-sum test (*p* < 0.001)

From comparison of genes near in vivo DNA binding sites for these B-ARRs at endogenous and elevated cytokinin levels, a TF-TF interaction network was constructed to analyze the redundant and diverged role of B-ARR family members (Fig. 2c). In addition, a directed gene regulatory network was constructed using the three cytokinin-treated ChIP-Seq results and publicly available DAP-Seq results^33^ as edges and changes in target gene expression (as measured by steady state RNA level) as nodes (Fig. 2d). While the *B-ARRs* are not themselves transcriptionally cytokinin responsive, 6-BA induced an increase in B-ARR binding of cytokinin “target genes” suggesting they regulate the expression of cytokinin responsive genes^11^. Individual B-ARR shared many targets between mock and 6-BA treatment datasets. For example, 85% (4075 of 4822 mock treated plants) of ARR10 mock targets overlap with 4 h 6-BA targets (Fig. 2c). All 10 *A-ARRs* (*ARRs 3, 4, 5, 6, 7, 8, 9, 15, 16*, and *17*), well-known cytokinin response genes, were among the targets of B-ARRs (Fig. 3a). At endogenous cytokinin levels, ARR1, ARR10, and ARR12 shared 503 targets (Fig. 3b). In addition, ARR1 and ARR10 shared another 1915 targets. In contrast, ARR12 shared less targets than either ARR1 or ARR10. At the elevated cytokinin levels (4-h 6-BA treatment), a set of 3373 targets were shared by ARR1, ARR10, and ARR12 (“common set of targets”) while 8770 targets were bounded by at least one of the three B-ARRs (“union set of targets”) (Fig. 3c, Supplementary Data 2). Additionally, ARR1 shared more targets with ARR10 than ARR12. The results indicated that many targets under mock treatment (endogenous cytokinin) were also bound by B-ARRs upon 6-BA treatments of 4 h or 3 days (Fig. 3d). However, the number of target genes increased from 2815, 5128, to 10,340 up 6-BA treatments (Figs. 2c and 3d). When genome-wide binding sites of recombineered ARR10_BA were compared to those generated using ChIP-seq of a tagged, over-expressing ARR10 by Zubo and colleagues^21^, an overall correlated profile of peak locations was found (Supplementary Fig. 3a). Compared to dataset 1 of Zubo et al.^21^ 2783 (69.5%) out of 4004 potential targets were also identified in our study (Fisher’s Exact test, *p*-value < 0.001, Supplementary Fig. 3b). If only the ARR10 “regulated targets” (those with evidence of transcriptional activity) in dataset 2 of Zubo’s study were considered, 87.4% were overlapping with our B-ARR union target set (Supplementary Fig. 3c). Though the recombineering experiment and the over-expressing experiment showed a high degree of agreement, our combined analysis of three B-ARRs revealed many additional in planta B-ARRs genomic binding locations (Supplementary Fig. 3d). The gene expression analyses were done using RNA-seq data from plants treated with 6-BA treatment for 4 h and using an *arr1/10/12* triple mutant (Supplementary Data 3 and Data 4). In total, 554 genes were differentially expressed in response to cytokinin treatment whereas the expression of 2323 genes were affected in the triple mutant (*q*-value <= 0.05 and 1.6-fold changes as cutoff, Supplementary Fig. 4a, b). When previous transcriptomic data and recent *CaMV 35S* over-expression ARR10 ChIP-seq data^21^ were compared, the expression of 813 common targets and 1713 union targets were observed to be affected by cytokinin treatment (Supplementary Data 5). These union target genes that showed cytokinin-induced expression changes were designated as “core target genes” of the B-ARRs. The top 50 genes ranked by their maximum peak scores contained eight known cytokinin biosynthesis/degradation or response genes (16%) including *type-A ARRs* (*ARR4, 5, 7,15*), cytokinin receptor (*WOL/AtHK4*), and cytokinin degradation enzyme (*CKX5*) and showing a 10-fold enrichment (*p* < 0.001, binomial test, Supplementary Fig. 5a, 5b). Finally, the intersection of 1713 core target genes and a previous large-scale TF binding dataset^33^ was used to construct a cytokinin transcriptional gene regulatory network (Fig. 2d, Supplementary Data 6), providing a framework for future studies of cytokinin response genes.

### A negative feedback loop in the cytokinin regulatory network

Previous indirect evidences including genetic studies, transcriptional profiling results, and promoter deletion analyses of *A-ARRs* suggested that these genes are targets of B-ARRs^20,34,35^. Our results show significant increase of B-ARR binding to the promoter of *A-ARRs* in response to cytokinin (Fig. 3a) and *A-ARR* genes are among the top-ranking targets of multiple B-ARRs (percentile ranking <5%). Upon exogenous cytokinin treatment (6-BA treatment for 4 h to 3 days), B-ARRs show significant increase of binding to *A-ARRs* (Fig. 3e) and their downstream target genes (Fig. 3f; Wilcoxon rank-sum test *p* < 0.001). In contrast, there was not much change in binding at the promoter of the *EIN3 binding factor (EBF2)* (Fig. 3e), a negative regulator in the ethylene signaling pathways^36^. Interestingly, a few *B-ARRs*, including *ARR1, ARR10, ARR12, ARR18*, and *ARR14* were among low-ranking targets (Fig. 3e). Overall these analyses support a scenario in which the promoters of the *A-ARR* genes are bound by B-ARRs representing an efficient feedback mechanism to fine-tune cytokinin responses in the plant.

### Functional classification of B-ARRs targets

**Fig. 4:**
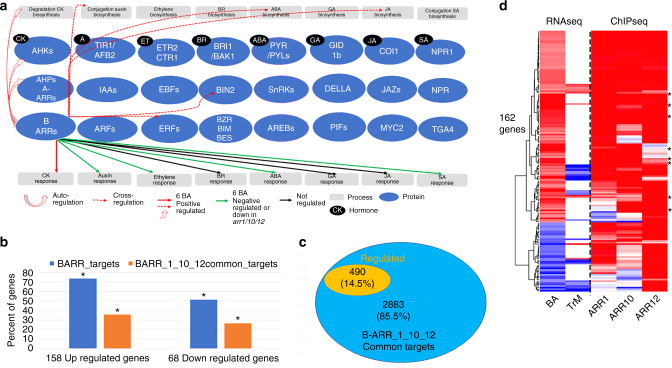
Enriched GO categories for B-ARRs target genes. **a** Interaction graph drawn from GO categories of nearby target genes of B-ARRs. The model shows that B-ARRs regulate every step of both cytokinin biosynthesis and signaling pathways and cross-regulate other plant hormone pathways. Hormone annotation legend: CK cytokinin, A auxin, ET ethylene, BR brassinosteroid, ABA abscisic acid, GA gibberellin, JA methyl jasmonate acid, SA salicylic acid. Red arrow: genes with increased transcript levels after cytokinin treatment; Green arrow: genes with decreased transcript levels in *arr1/10/12* triple mutants compared to wild type or genes repressed by cytokinin. Black arrow: genes with regulated (for BR) and not-regulated (for GA and JA) transcript levels in *arr1/10/12* triple mutants compared to wild type. **b** Comparison between B-ARR targets and cytokinin response genes in the golden list^20^ using Fisher’s exact test (asterisk (*) indicates corrected *p* < 0.05). **c** The subset of B-ARR 1, 10, and 12 candidate target genes also showing transcript changes (regulated). **d** A heatmap shows both cytokinin regulation and B-ARR binding for a subset of 162 common targets of B-ARR 1, 10, and 12. *A-ARRs* were highlighted (*). *Z* scores for RNA-seq were based on log2 fold changes (6-BA treatment compared to mock and triple mutants compared to wild type) and for DNA binding were based on log2 peak score (−log2(*p*-value)). RNA-seq was acquired from 4-h 6-BA treatment (BA) or in *arr1/10/12* triple mutants (TrM). DNA-binding for 6-BA treatment target genes of B-ARR-1 (ARR1), B-ARR-10 (ARR10), and B-ARR-12 (ARR12)

A previous study of binding sites for the master transcriptional regulator for the plant hormone ethylene revealed major feedback loops where EIN3 directly targeted almost all essential genes in the ethylene signaling pathway, as well as key regulators of other phytohormone pathways^36^. To find out whether this TF-governed auto-regulation and cross-regulation with other pathways also holds true or not for B-ARRs, we performed gene ontology (GO) analysis of target genes of B-ARRs using the top 3000 genes ranked by IDR score^37^. These analyses revealed enrichment for similar biological function and processes for all tested B-ARRs (Supplementary Fig. 6). GO enrichment analysis was consistent with the fundamental and diverse role of cytokinin (Supplementary Fig. 6). B-ARR DNA binding sites are highly associated with plant hormone responsive genes and cytokinin genes (GOTERMs: response to plant hormone stimulus *p*-value 1.5E-26, two-component signal transduction *p*-value 6.3E-15). Among the 3373 common targets of the three B-ARRs (ARR1/ARR10/ARR12) tested, we observed enrichment of hormone-related genes (Fig. 4a, Supplementary Data 7). Additionally, the top 3000 genes ranked by ARR10 peak scores were used to refine the GO analysis. The top GO terms are similar in the global analysis for the targets of three B-ARRs. These targets of B-ARRs include the primary cytokinin response genes, *A-ARRs* and the cytokinin receptor *AHK4*. Although B-ARRs are not regulated at the transcriptional level by cytokinin, it is interesting that several (*ARR1, ARR10, ARR12, ARR14,*and* ARR18*) were found in the B-ARR target gene list as being under control of *B-ARRs*. In addition, B-ARRs were found to bind at cytokinin biosynthesis and degradation pathway genes (Fig. 4a, Supplementary Data 7). Thus, the transcriptional responses directed by B-ARRs may include nearly every step from cytokinin perception and signaling transductions to the TFs. Like EIN3, the response of B-ARRs to cytokinin may involve cross-regulation with other plant hormone biosynthesis, signaling, and response pathways (Fig. 4a). B-ARRs were found to target the auxin receptor genes *TIR1* and *AFB2*, as well as the auxin transportation efflux carrier genes, the *Pin-formed *and* Pin-formed like (PIN3/4/7)*, and the *GH3s* genes which mediate auxin conjugation. Auxin transcriptional response regulators, including several *ARFs* and many *Aux/IAAs* genes, were also among the list of B-ARR targets (Fig. 4a, Supplementary Data 7). The most striking features among the hormone-related targets of B-ARRs are genes encoding the master transcriptional factors such as *MYC2, PIFs, BES/BZR,*and *ERFs* (Fig. 4a), each responsible for mediating the transcriptional responses to other plant hormones^38–42^. B-ARRs also target genes encoding plant hormone receptors such as *BRI1/BAK1/BAK7*^43^, *PYR/PYLs*^44^, and *TIR1/AFB2*^45^ (Fig. 4a). In addition, B-ARRs targeted the plant hormone negative signaling component genes, such as *Aux/IAAs* for auxin^46^, *EBF1/2* for ethylene^47^, *BIN2* for brassinosteroid^43,48^, and the DELLA protein *GAI* and *RGA* for gibberellin^49^.

Next, we inquired into whether the transcription of B-ARR target genes are regulated by cytokinin levels (Supplementary Data 3 and Data 4). The regulated genes were then layered into plant hormone pathways targeted by B-ARRs (Supplementary Data 7). Cytokinin treatment increased gene transcripts for most *A-ARRs*, *AHK4*, and cytokinin degradation enzymes in the cytokinin pathways (Supplementary Data 7). Whereas gene transcripts were shown to decrease for important negative regulators of other hormone pathways, including several *IAAs*, and *GH3s* for auxin, *SnRK3.14* and *NRT* for abscisic acid (ABA), as well as modification enzyme for salicylic acid (SA) (Supplementary Data 7). In addition, in the triple mutant background, the auxin receptor *AFB2* and the auxin efflux carrier *PIN7* were down-regulated (Supplementary Data 7, gene names underscored) whereas *TAS3* and the ethylene receptor *ETR2* were up-regulated (Supplementary Data 7, gene names in green). These results suggest that cytokinin pathway TFs target important regulators of other plant hormones pathways, potentially leading to diverse outputs for developmental and growth programs, as well as responses to environmental cues.

Numerous studies have reported that TF binding does not necessarily coincide with changes in gene transcription^50^. In the case of EIN3, only 30% of the ET-induced binding events were associated with transcriptional changes^36^. We examined genes in the “golden list” for cytokinin responses which were identified by Bhargava^20^ using a meta-analysis of multiple expression datasets, to determine the overlap with genes that we identified as B-ARR targets. In all, 116 (73.4%) of 158 up-regulated genes in the golden list were identified among the 8770 union binding targets of three B-ARRs, suggesting significant association between B-ARR binding and cytokinin-induced transcriptional responses (Fig. 4b; Fisher’s Exact Test *p* < 0.001). When compared to the 3373 common targets of three B-ARRs, the percentage of overlap dropped to 35.4% which is still highly significant (Fig. 4b; Fisher’s Exact Test *p* < 0.001). Similarly, 35 (51.4%) of 68 down-regulated genes in the golden list were overlapped among the 8770 union targets of B-ARRs (Fisher’s Exact Test *p* < 0.001) although only 26.5% of these were among the 3373 common targets of the three key B-ARRs (Fig. 4b; Fisher’s Exact Test *p* < 0.001). A small portion (74 of 226 or 32.7%) of genes in the golden list were not found in the list of B-ARRs targets. They might be either indirect cytokinin response genes or the targets of other B-ARRs that were not tested in this experiment. It is interesting that activators like B-ARRs directly target cytokinin repressed genes which may involve the recruitment of other co-regulators. Only 490 (14.5%) of 3373 common targets of three B-ARRs (ARR1/ARR10/ARR12) were affected transcriptionally either in triple mutant or by cytokinin treatment (Fig. 4c). A subset of 162 common targets that the binding of B-ARRs responded to cytokinin treatment also changed their expression upon cytokinin treatment (Fig. 4d, Supplementary Fig. 4c). When another dataset of cytokinin response genes was used^51^, 82% of overlap between the expressed gene list and our ChIP-seq data were observed (Fisher’s Exact Test, *p* < 0.001).

### Switch of B-ARR motif specificity in response to cytokinin

**Fig. 5:**
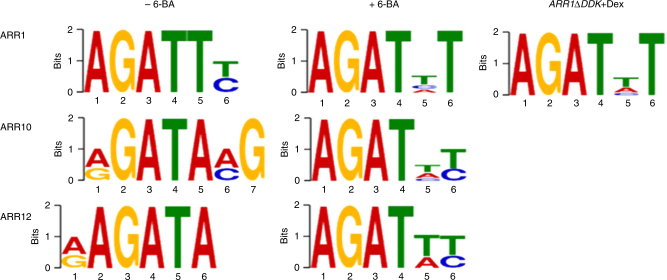
Identification of the 6-BA dependent B-ARR binding motif. Similar DNA binding motifs of B-ARRs become nearly identical (B-ARR-6-BA motif) in response to increased cytokinin treatment (6-BA). AGAT is the core binding-motifs of B-ARRs and AGATHY, H(a/t/c), Y(t/c) are the B-ARR-6-BA motifs after cytokinin treatment. −6-BA: DMSO, +6-BA: 10 µM 6-BA. Inducible *ARR1∆DDK* was treated using 10 µM dexamethasone (*ARR1∆DDK* + Dex)

The targets of B-ARRs are predicted to be enriched for specific binding site motifs. We used the top 1000 conservative narrow peaks from each B-ARR, ranked by the IDR peak score and included two hundred bps flanking the peak summits, for motif calling using the MEME-ChIP suite (Fig. 5). Analyses of the identified B-ARRs motifs suggested several important conclusions. First, without any treatment, B-ARRs binding motifs are very similar to each other; each contains an AGAT core flanked by degenerate sequences with the first A in the core being slightly more degenerate (A/G). Although the motifs are similar, there are also significant differences that can be distinguished by the degenerate sequences (Fig. 5). These findings are consistent with previous in vitro binding and the PBM assays, identifying AGAT as the core B-ARR binding motif^52^. Second, after cytokinin treatment, all binding motifs strikingly become invariant AGAT((t/a/c)(t/c)) which we have termed the B-ARR-6-BA motif (AGATHY) (Fig. 5). In order to better understand the 6-BA-dependent change of the core binding motif, we additionally analyzed the genome-wide binding profile of a truncated version of *ARR1* (*ARR1∆DDK*). This *ARR1* version does not possess the receiver domain and represents a constitutive active form of ARR1^15,18^. ChIP-seq and MEME analysis of ARR1∆DDK uncovered the B-ARR-6-BA motif as the primary binding motif (Fig. 5-*ARR1∆DDK* + Dex), indicating that cytokinin can directly affect B-ARR binding through the receiver domain. Although previous in vitro-derived B-ARR binding-motifs were identified by Weirauch et al.^53^, our study reveals that the subfamily-1 B-ARRs share a similar DNA-binding motif and provides direct in vivo evidence of the DNA-binding signatures of B-ARRs. The similarity between 6-BA-treatment-specific DNA binding signature of B-ARRs and that of the constitutive active ARR1∆DDK provides additional insight into the in planta mechanism by which cytokinin modulates TF function. The B-ARR-6-BA motif was enriched in the promoter regions (−1.5 k to + 100 bps surrounding the TSS of 83% (3334/4012) of ARR1 bound genes compared to the whole genome background (Fisher’s Exact Test, *p* < 0.001, Supplementary Fig. 3e). In the Zubo et al. study, 85% (687/804) of the ARR10 “regulated target” genes also showed enrichment of the B-ARR-6-BA motif (Fisher’s Exact Test, *p* < 0.001, Supplementary Fig. 3e). While the B-ARR-6-BA motif is commonly present in 25,188 (75%) of all promoters in the Arabidopsis genome, only 3334 (13%) of these were bound by ARR1, suggesting that the motif is not sufficient to mediate the transcriptional response to cytokinin. In addition, we compared the ratio of TF binding sites identified in the 5′ and the 3′ (Supplementary Data 8). In the top 5% target genes examined, there was a 2-fold enrichment of (5′/3′) which dropped to 1.8-fold in the top 10% target genes and 1.7-fold for binding sites relative to all genes. There was also preferential binding at 5′ regions of the B-ARRs, which was consistent with the binding profile of other cytokinin regulated genes (Supplementary Data 9). These finding reveal that the top-ranking target genes have slightly preferable binding at their 5′ as opposed to 3′ but there are many ChIP-seq peaks (potential regulatory elements) present at gene 3′ ends as well.

### B-ARRs target to WUS in stem cell maintenance

Cytokinin is a central player in shoot apical meristem initiation and maintenance^22,23^. The fact that the *arr1/10/12* triple mutant produces a smaller size shoot apical meristem also implies that the cytokinin transcriptional responses are important for stem cell maintenance^8^. It is possible that cytokinin signaling directly targets either *WUS* or *CLV3*, genes that control the meristem size, the *WUS-CLV3* loop^54,55^. Interestingly the *WUS* gene which plays an important role in shoot apical meristem maintenance, a cytokinin-dependent process^22,55^, was a consistent low-ranking target of B-ARRs. We further explored dynamic binding of B-ARRs at the promoter of *WUS* and identified the B-ARR binding site within the promoter of *WUS*. After 4 h of cytokinin treatment, clear binding of ARR1 at the *WUS* promoter could be observed which became even more apparent in the 3-day hormone treatment samples (Fig. 3e). Thus, *WUS* is likely a, cytokinin-dependent, target of ARR1 (Fig. 3e). Similarly, cytokinin-induced binding at the promoter of *WUS* was also found for ARR12, showing a significant increase with hormone treatment and ARR10 targeting the promoter of *WUS* at both mock and cytokinin treatment conditions, although with less binding (Fig. 3e).

**Fig. 6:**
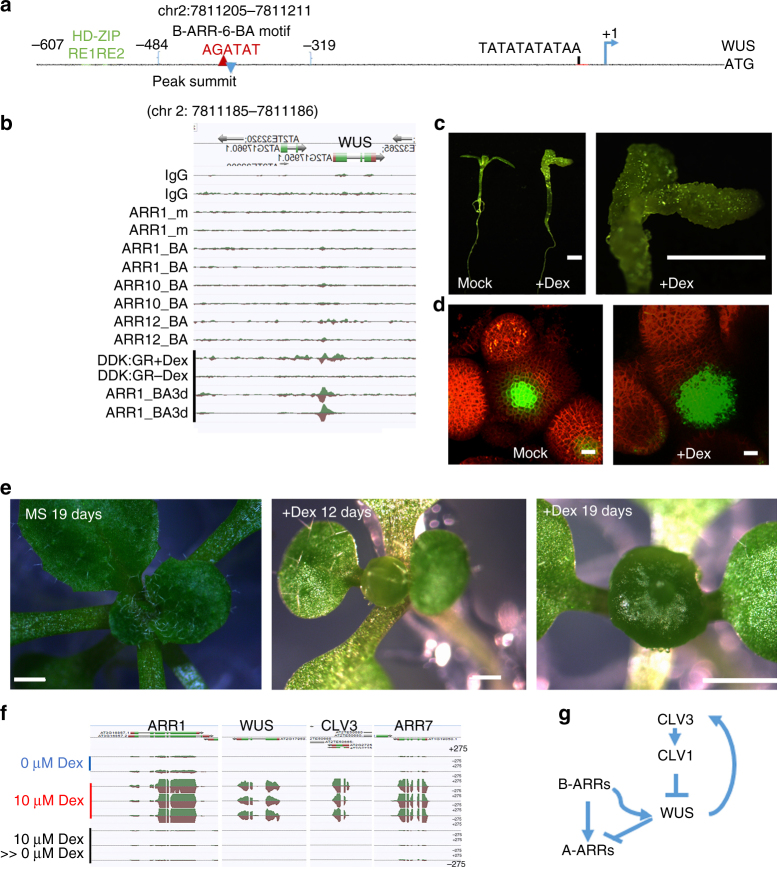
Cytokinin dependent B-ARRs targeting of the *WUS* promoter. **a** The location of the AGATAT B-ARR-6-BA motif relative to the *WUS* gene’s TSS. The number (7811185) of the peak summit is the genomic position on the second chromosome. **b** An AnnoJ snapshot showing B-ARRs binding to *WUS* promoter requires a high concentration of cytokinin. *Y*-axis heights are scaled to same value. Black bar indicates plants grown on plates with 10 µM 6-BA for 3 days (BA3d) or *ARR1ΔDDK::GR* plants treated with 10 µM Dex. **c**, **d** Phenotypes of *p35S::ARR1ΔDDK::GR*. **c** Phenotypes of 11 days old transgenic plants without Dex (Mock), with Dex (+Dex), and at higher magnification (+Dex). Scale bar = 1 mm. **d** 3D-reconstructed views of shoot apical meristems (SAMs) showing *pCLV3::mGFP5-ER* (CZ/stem cell reporter; green) and *35S::YFP29-1* (plasma membrane-localized YFP marks the outlines of all cells, red). SAM of mock-treated plants 4 days after treatment (Mock) and SAM of Dex-treated plants 4 days after treatment (+Dex). Scale bar = 10 µM. **e** Enlarged shoot apical meristem (“ball” of SAM)) by the inducible expression of the constitutive active form *ARR1* (*ARR1∆DDK:GR*) in stem cell domain (*CLV3* domain). Scale bar = 1 mm. The transgenic plants without Dex induction as a control after growth on MS for 19 days (MS 19 days). “Ball” of SAM after growth on 10 µM Dex plate for 12 days (+Dex 12 days). “Ball” of SAM after growth on 10 µM Dex plate for 19 days (+Dex 19 days). **f** RNA-seq reads tracks of the “ball” of SAM system demonstrating the expression of *ARR1, ARR7*, *WUS*, and *CLV3*. Transgenic plants grown on MS for 19 days (0 µM Dex), on 10 µM Dex plate for 19 days (10 µM Dex) and grown on 10 µM Dex plate for 12 days then transferred to MS for 7 days (10 µM Dex > 0 µM Dex). All tracks have the same scaling of *y*-axes (+275/−275). **g** A sketch showing the interface between the cytokinin response and the *CLV3-WUS* circuit

Two lines of evidence point to *WUS* as a candidate target of cytokinin TFs, such as the B-ARRs. First, cytokinin signaling revealed by two component sensor (TCS) is higher in *WUS* domain than any of the other domains in shoot apical meristem^56^. Second, cytokinin induces expression of a *WUS* transcriptional reporter gene^22^. Interestingly, as mentioned above *WUS* consistently appears as a low-ranking target gene in ChIP-seq analyses for each of the tested B-ARRs in plants not exposed to exogenous cytokinin. One possibility is that binding of B-ARRs to the promoter of *WUS* requires an elevated level of cytokinin. When plants were grown with cytokinin, we identified a single binding site in the *WUS* promoter with the B-ARR-6-BA motif (AGATAT) located at the peak summit (Fig. 6a) along with increased binding (Fig. 6b black bar). These findings are consistent with a previous report that activation of transcriptional reporter *pWUS:GFPer* in the shoot apical meristem requires high (1 mM) concentrations of cytokinin^22^. Since a high concentration of cytokinin was required to activate *WUS*, the effect had been previously thought to be indirect. However, our results demonstrate the presence of a B-ARR-6-BA motif in the *WUS* promoter, implying that *WUS* may be an in vivo target of B-ARRs. Interestingly, the B-ARR-6-BA motif is adjacent to HD-ZIP response elements, suggesting possible interactions with other TFs that may be required for stem cell maintenance in the shoot apical meristem (Fig. 6a). Indeed, recent reports provide evidence for the interaction between B-ARR and HD-ZIP^21,57–59^.

To understand the functional significance of B-ARRs-mediated TF binding and increased *WUS* gene expression, a constitutively active form of *ARR1* was introduced into plants using a Dex inducible system^60^. Deletion of the signal receiver domain *(∆DDK) of ARR1* (a *B-ARR*) has been shown to constitutively activate cytokinin signaling by unmasking the transactivation function of ARR1^15^. The *ARR1* deletion construct was fused to the glucocorticoid-inducible artificial transcription factor (GR) and expressed from the cauliflower mosaic virus (*CaMV*) *35S* coat protein gene promoter to generate dexamethasone (Dex) inducible *35S:ARR1∆DDK:GR*^15^. In the absence of dexamethasone (Dex), transgenic plants carrying the *35S:ARR1∆DDK:GR* construct were phenotypically normal (Fig. 6c, Mock). Upon dexamethasone induction, these plants displayed a phenotype similar to tissue explants propagated on cytokinin culture medium (Fig. 6c, +Dex). If B-ARR binding at the B-ARR-6-BA motif in the promoter of *WUS* activates *WUS* transcription, then, based on current knowledge of this pathway, transcriptional activation of *CLV3* is subsequently expected^54,55^. To test this model, the *35S:ARR1∆DDK:GR* construct was introduced into plants carrying, *pCLV3:mGFP5-ER* (*CLV3* promoter driving the expression of endoplasmic reticulum-localized green fluorescent protein), a fluorescent reporter for stem-cells and *35S::YFP29-1* (ubiquitous promoter driving the plasma membrane-localized yellow fluorescent protein) and marker for cell boundaries which allows visualization of all SAM cells. We observed that constitutive activation of cytokinin signaling led to expansion of stem-cell domain marked by the *pCLV3* reporter upon induction of *ARR1∆DDK:GR* (Fig. 6d). An alternative explanation for the activation of the *pCLV3* reporter would be due to increased transcription of *A-ARRs* by the *35S:ARR1∆DDK* as a previous study reported the requirement of *ARR7* and *ARR15* to maintain *CLV3* expression^23^. However, the size of shoot apical meristem in *35S:ARR1∆DDK:GR* system did not increase. We then tested the effect of expression of *ARR1∆DDK* under control of the *pCLV3* promoter using *pCLV3:LhG4/pMX6xOPs:ARR1∆DDK:GR*, a two-component inducible system. An enlarged shoot apical meristem (“ball” of SAM) was observed. When expression of the transgene was induced by Dex treatment, all ten characterized lines harboring both *pCLV3:LhG4* and *pMX6xOPs:ARR1∆DDK:GR* showed similar phenotypes at the seedling stage (Fig. 6e, +Dex 12 days and + Dex 19 days). Plants were first grown on MS plates and then transferred to 10 µM Dex plate for induction, phenotypes observed in adult transgenic plants included a “ball’ of the primary SAM (Supplementary Fig. 7a), indeterminate floral meristem (Supplementary Fig. 7a), and a “ball” of the lateral SAM (Supplementary Fig. 7a, “Ball” of Lateral SAM). The observed “ball” of the SAM phenotype is reminiscent of the “dome” shape of SAM in transgenic plants observed when *WUS* is expressed under the control of the *pCLV3* promoter^61^. The expression of *WUS* in *pCLV3*:LhG4/pMX6xOPs inducible system was also reported to have an enlarged shoot apical meristem^62^. Transgenic plants germinated on MS plates and then transferred to 10 µM Dex plates, both *pCLV3:LhG4/pMX6xOPs:WUS:GR* and *pCLV3:LhG4/pMX6xOPs:ARR1∆DDK:GR* also showed a similar “ball” of SAM phenotype (Supplementary Fig. 7b). RNA-seq of “ball” cells isolated from the SAM clearly showed *ARR1∆DDK* was over-expressed compared to the full length of *ARR1* (Fig. 6f). Moreover, transcriptional activation of both *WUS, and CLV3* was observed (Fig. 6f) although *CLV3* was not found to be a target of any of the tested B-ARRs. These results are consistent with the hypothesis that B-ARRs activate *WUS* expression, which in turn activates *CLV3*. Therefore, our results provided a link between the cytokinin transcriptional response and the *WUS-CLV3* circuit in the shoot apical meristem (Fig. 6g).

## Discussion

In this study, we employed recombineering to engineer a Ypet-tag onto three B-ARRs, ARR1, ARR10, and ARR12. This system enabled monitoring of endogenous expression and protein localization patterns for three B-ARR TFs. It also allows real time visualization of the cytokinin primary TFs which was previously only done by a GUS fusion. The recombineered B-ARRs were also used for ChIP-seq experiments, allowing the identification of in vivo binding sites and putative target genes. Comparison of binding profiles from both endogenous and elevated cytokinin conditions showed marked differences in both motif sequence and target gene number. Based on these dosage experiments, there may be as many as 10,000 cytokinin response genes in the *B-ARR* network. Thus, a limiting factor might be the amount of endogenous cytokinin to modify B-ARRs.

The genome-wide identification of the targets of B-ARRs provides a new resource to understand how cytokinin may regulate diverse plant growth and developmental processes, as well as respond to stresses in conjunction with other phytohormones at different regulatory layers, such as biosynthesis, transportation, perception, or signal transduction. The tissue/cell expression patterns of *ARR1*, *ARR10*, and *ARR12* are quite similar^29^. Similarly, in vivo ChIP-seq targets for the three B-ARRs also resemble one another. When cytokinin levels are elevated, B-ARRs target many plant hormone negative regulators, such as *A-ARRs*^63^, *Aux/IAAs*^64^, and *EBFs*^47^. Targeting of multiple negative regulators in multiple hormone pathways might provide a quick and effective avenue to abate this hormone imbalance ensuring quick re-equilibration of responses. Additionally, a recent ChIP-seq study using tagged, over-expressed *ARR10* identified a set of ARR10-bound cytokinin responsive genes^21^. Since over-expression of *ARR10* was able to rescue *arr1/10/12* triple mutants^21^, it is not surprising that the list of targets (81.5% or 3265 out of 4004 genes) shows a statistically significant overlap with the targets that we identified using recombineered ARR genes (Supplementary Fig. 3b, Fisher’s exact test, *p* < 0.001). The ARR10 over-expressing ChIP-Seq results shared 2783 gene targets with our ARR10_BA and had 1221 unique targets not identified by ARR10_BA. Interestingly, the 2783 overlapping target genes found by both studies had higher peak scores than unique target genes (Supplementary Fig. 8a), suggesting that these are high confidence ARR target genes. However, expression of the native level of the *ARRs* using recombineered genes may allow the identification of more authentic, cytokinin-response relevant target genes. In this regard, dataset 1 of Zubo et al. contained 1221 additional targets that did not show enrichment for any meaningful GO terms while 3489 targets unique to the recombineering ARR10 ChIP-seq data showed significant enrichment for plant hormone GO terms (Supplementary Fig. 3b). Moreover, over-expression of ARR10 identified known target gene for other B-ARRs (Supplementary Fig. 3b and 3d; Fig. 8b). However, the number of unique targets dropped to 739 when we compared these binding sites to ChIP-seq results for other B-ARR 6-BA treated tissues (Supplementary Fig. 3b, 3d). Moreover, the 482 targets shared between other recombineered B-ARRs and ARR10 over-expressing line had lower peak scores compared to the 2783 shared ARR10 target genes between our study and Zubo et al. (Supplementary Fig. 8b), suggesting potential off-target binding in ChIP-seq experiments using ARR10 over-expressing plants. Combined, these studies provide biochemical evidence confirming genetic redundancy among B-ARR factors (Supplementary Fig. 3d). Interestingly we also uncovered a change of the B-ARR binding motif upon cytokinin treatment which was likely missed in earlier studies since this observation requires comparing B-ARRs at both endogenous conditions and cytokinin treatment (Fig. 5). Finally, gene targets identified by over-expression of ARR10 by Zubo et al^21^. identified a number of the top-ranking B-ARR targets in our study (Supplementary Data 10). Thus, our findings provide unique information not available in previous studies, providing novel insight about full response of the plants to cytokinin.

Although several types of in vitro experiments identified potential DNA binding motifs for B-ARRs, the in vivo binding sites identified here provided a unique opportunity to further analyze DNA binding events. Cytokinin promoted B-ARR motif switching, from a more degenerated motif to a canonical B-ARR-6-BA motif (AGATHY). B-ARRs are not regulated at transcriptional level by cytokinin but are post-transcriptionally modified^11,13^. One possibility is that without such modification B-ARRs only loosely bind their targets; further studies are necessary to explore the impact of phosphorylation on the motif site selection. ARR1 showed the highest cytokinin-dependent enrichment of binding to its targets. Without cytokinin treatment, only 2815 potential targets were identified but the number increased to 5128 after just four hours of cytokinin treatment, and it further increased to 10,340 targets when treated for 3 days. These targets could be further arranged in a hierarchical manner into early binding (targets found in both mock and 4-hour BA treatment as 1st), short BA treatment binding (targets found in both 4-hour and 3-day BA treatment as 2nd), and longer BA treatment binding (targets found only in 3-day BA treatment as 3^rd^) (Supplementary Fig. 3f). We speculate that longer cytokinin treatment may change chromatin structures such that more binding sites become available. Although the high dose and 3-day treatment may potentially result in false positives peaks, the high overlap between lower level hormone treatments and these high-dose experiments confirm the relevant cytokinin responsiveness. This cytokinin-dependent binding of ARR1 to its targets might be explained by hormone-dependent alteration in ARR1 protein stability. Alternatively, a high concentration of cytokinin may trigger the phosphorylation cascade resulting in activation of TF B-ARRs, and these phosphorylated B-ARRs may bind their targets more tightly than those lacking the modification. In this two-component multiple phosphorelay system, it is thought that phospho-activated B-ARRs change the conformation of their receiver domain^15,18^. The model asserts that B-ARRs constitutively occupy their binding sites, only becoming “active” upon phosphorylation^65^. Our time-series ChIP-seq analysis provides correlative evidence that cytokinin increases both binding of B-ARRs to their targets and the number of targets bound. However, this suggestion must be further addressed by complementary experiments, such as the reduction of endogenous cytokinin levels using a regulated expression of *CKX3*^66^ to examine the impact of endogenous cytokinin removal on the interaction between B-ARRs and their targeted promoters. Alternatively, using mutations at the conserved phosphorylation sites in B-ARRs may also help to test this model^67^.

Importantly, we identified a B-ARR binding site in the promoter of *WUS* gene, which encodes a homeodomain TF that was shown to repress a subset of A-ARRs^24,55^. *WUS* has been suggested to work in conjunction with the cytokinin pathway^22,24^ to establish a stem cell niche ensuring early embryogenesis and later for maintenance of the shoot apical meristem^55^. The study using the new reporter revealed that the *WUS* expression domain overlaps with SAM regions where cytokinin activity is the highest^68^. Similarly, the *pWUS::GFP-er* reporter can be activated in SAM by cytokinin but only at a high concentration of cytokinin. However, subsequent (expected) activation of a *pCLV3::GFP-er* reporter was not observed^22^. A previous explanation of why *WUS* does not robustly respond to low cytokinin levels was that the expression of *WUS* was only in the a specific (*WUS*) subdomain of the SAM and that the cytokinin was not accessible to the *WUS* domain. Our results indicate that most of the potential binding motifs of B-ARRs in the promoter region of *WUS* were unoccupied. However, we identified one B-ARR-6-BA motif, located precisely at the peak summit of the conservative narrow peak, in multiple B-ARRs data sets. The identification of a strong in vivo B-ARR DNA binding site within the promoter of *WUS* that requires a high level of cytokinin, provides new direct evidence for this association. In addition, the B-ARR-6-BA motif (−420bp) was found to be within 57 bps as two HD ZIP response elements (−540 to −565) that were previously identified as *WUS* regulatory sites^69^. It is possible that the coordination or competition among these TFs is a feature of *WUS* regulation, which is itself controlled by a *WUS-CLV3* negative feedback loop, keeping the meristem size constant in each species. Moreover, the activation of *WUS* by B-ARRs downstream of cytokinin signaling should also be tightly attenuated in the shoot apical meristem. When a constitutive active form of *ARR1* was introduced, removing the possibility of feedback inhibition, the activation of *WUS* led to an expansion of stem cell domain. Interestingly, ectopic expression of *ARR1∆DDK* did not lead to an enlarged SAM, indicating the possible presence of an unknown inhibition mechanism from other domains within the SAM. Expression of *ARR1∆DDK*, under the control of *pCLV3* promoter, resulted in an enlarged shoot apical meristem (“ball” of SAM). This phenotype may result from activation of *WUS* by B-ARRs in the stem cell that cannot be dampened. Since *type-A ARRs* were highly activated by B-ARRs, increasing the level of *type-A ARRs* may cause increased expression of *CLV3* as *ARR7* and *ARR15* are required for the expression of *CLV3*^23^. However, as negative regulators, the inhibition mechanism of *type-A ARRs* to cytokinin signaling remains unknown^11^. Similarly, the activation mechanism of *type-A ARRs* leading to *CLV3* expression is also not clear. If *CLV3* is the solely interface between B-ARR and A-ARRs leading to activate of *CLV3*, then transcription of *WUS* would be expected to shut down.

The “ball” of the SAM phenotype and high levels of both *WUS* and *CLV3* expression observed in *pCLV3:ARR1∆DDK* system suggests that B-ARRs activate the transcription of *WUS*, then activate *CLV3* leading to expand the shoot apical meristem, resulting in the “ball “of SAM. Previous reports of *pCLV3:WUS* expression resulted in similar phenotypes, further providing functional relevance to our model^61,62^. This result is also consistent with the idea that multiple feedback loops exist in the shoot apical meristem to adjust its size^22^. The introduction of a mutation in the B-ARR binding site in the promoter of *WUS* would test the idea that *WUS* is, at least in part, under direct transcriptional control of B-ARRs. Taken together, these findings provide new insights to the role of the cytokinin transcriptional responses in stem cell maintenance.

## Methods

### Plant growth conditions

Three-days-old seedling tissue was collected for these experiments unless otherwise noted. Seeds were surface-sterilized and sown on agar plates (1.8%) containing Murashige and Skoog salts (pH 5.7) and 1% sucrose. Seedlings were grown vertically for 3 days at 22 °C under long day condition using a 16-h light/8-h dark cycle. The seedling was subsequently treated with liquid Murashige and Skoog medium (MS) pH 5.7 with 0.08% Silwet 77 containing 10 µM 6-BA (in DMSO) or mock treated with an equivalent volume of DMSO. The three-days continuous hormone treatment was orchestrated by germinating the tagged lines on MS with 10 µM 6-BA or MS with the same amount of DMSO as mock. 10 µM 6-BA was chosen based on the range from 1 µM to 20 µM cytokinin treatment^20^.

### Gene constructs and generation of tagged B-ARRs

Based on recombineering techniques^28^, a Ypet (yellow fluorescent protein) tag was recombineered into a transformable bacteria clone (TAC) clone such that the B-ARR gene was located at the center of the large insert clone. We employed the two-step recombineering method using the Flapase–Fret system. Positive Ypet clones were obtained first by selection against ampicillin as the insert contained an ampicillin resistant gene. This ampicillin marker was later removed. While the technique leaves a small scar between Ypet and the B-ARRs the method is easier than the GalK system^28^ and the scar serve as a linker between the tag and the B-ARRs. The Ypet gene was added at the C-terminus of each B-ARR gene of the whole B-ARR family but only ARRs 1, 10, 12, 11, 13, and 14 were successful. ARR1 was tagged using isoform ARR1.2 since the previous expression data indicated ARR1.2 as the major splice variant. The tagged TACs were sequenced to confirm sequence fidelity of the B-ARRs, the junction, and the fusion to Ypet within TAC. The tagged TAC clones were transformed into *GV3101* strains and transformed wild type *Columbia-0* plants using the flower dipping method^70^. After screening for Basta resistance, putative tagged lines were identified by PCR using forward recombineering test primer and reverse Ypet primers. The PCR products were gel-purified and sequenced to confirm the in planta tagging junction. The expression of the tagged B-ARRs (ARR1, ARR10, and ARR12) was then examined under Zeiss confocal microscopy 710 (Zeiss) using identical settings (6% laser power; master gain: ch1 910, ch2 248; digital gain 1, digital offset 0; pinhole 281 µM; filter ch1 521-546).

The active form of ARR1 was constructed using a deletion of the receiver domain of *ARR1* that was previously described^15,18^. The deletion called *ARR1∆DDK* was cloned into *pENTR D/TOPO* with *GR* fusion at the C-terminus and then the entire *ARR1∆DDK:GR* gene cassette was moved into the *pEG104* transformation vector to be in frame of a N-terminus *YFP*^71^. The *pENTR D/TOPO: ARR1∆DDK:GR* was cloned into *pMX6xOPs* vector and then the transgenic line was combined with the *pCLV3:LhG4* line to achieve stem cell-specific expression of *ARR1∆DDK*.

### Observation of phenotypes using the inducible system

The two-components inducible system was used^62^. The *pCLV3:LhG4* line was in *Landsberg erecta* (*Ler-0*) background. The *pMX6xOPs:ARR1∆DDK:GR* was transformed into Ler-0 background. The *pMX6xOPs:ARR1∆DDK:GR* lines were screened using gentamicin. Ten individual lines were used to cross with the *pCLV3:LhG4* line and were tested on MS medium with 10 µM Dexamethasone (Dex). MS medium with the same amount of ethanol was used as control. All ten lines showed the enlarged apical meristem phenotype. Initial observation of phenotypes was done by germinating seeds directly on Dex plates. The *pCLV3:LhG4/pMX6xOPs:WUS:GR* line was provided by Dr. Reddy’s lab and the same induction method was used.

### Chromatin preparation and immunoprecipitation

Seedlings were dried by paper towel and transferred in 1% formaldehyde solution. Cross-linking occurred under 5-10-5-min vacuum cycles with a quick vacuum release in between each cycle. A final concentration of 125 mM glycine was applied for 5 min to deactivate the remaining formaldehyde. Cross-linking resulted in translucent seedling tissue. Tissue was then liquid nitrogen cooled and either stored in −80 °C degree or directly ground and an extraction of chromatin was performed as previously described^36^. Chromatin immunoprecipitation (ChIP) was performed as previously described^36^ with modifications, including the use of Bioruptor sonicator (Diagenode, Belgium). Bioruptor settings used were: Low, 10 cycles of 25 s on, 120 s off. Sonication was performed in auto-cooling system with water bath at 4 °C. A small amount of chromatin (10 µl) was evaluated for shearing; the size range of chromatin was 150–450 bps, most fragments at 250 bps.

The commercial anti-GFP antibody (Thermo Fisher Scientific, A11111) was used for the immunoprecipitation reactions. Five microgram antibody and 50 µl of Dynabeads M-280 Sheep anti-Rabbit IgG (Thermo Fisher Scientific, catalog #11204D) were consumed for each reaction. The incubation was performed using previously described buffer^36^ at 4 °C overnight (16 h). The Dynabeads were washed using a low stringent wash buffer followed by a high stringent buffer^36^, a quick rinsing, and 5 min rotating at 4 °C. A final wash buffer was applied to clear the detergent from previous buffers followed by another 5 min rotating. During each wash, the tubes were quickly centrifuged before returning to the magnetic stand and trace amounts of buffer were removed to avoid non-specific binding carryover. The resulting ChIP DNA was collected in two elution buffers (100 µl each) at 65 °C and combined. The proteinase K digestion occurred at 55 °C. The reverse of cross-linking was done at 65 °C overnight (16 h). The ChIP DNA was then purified by extraction using phenol:chloroform:isoamyl alcohol (25:24:1) (Sigma, p3803) twice in a phase lock gel system before the ethanol precipitation step using glycogen for the pellet observation. The pellets were then washed with 70% cooled ethanol and dried in a speed-vac. The ChIP DNA was then dissolved in 50 µl water for subsequent library preparation.

### ChIP-seq library generation and sequencing

The single-end read libraries were prepared using the TruSeq ChIP kit from Illumina (catalog ID: IP-202-1024), with DNA size selection made with lab-made Serapure magnetic beads. Library concentration were assayed using the Qubit 2.0 Fluorometer (Thermo Fisher Scientific) and normalized to 1.7 Nano gram per microliter. Six multiplexed libraries were sequenced per lane on a HiSeq 2000.

### ChIP-seq data analysis

ChIP-seq data analyses were carried out using a suite of pipelines developed in our lab to run the alignment and analysis. Briefly, alignment was performed using Bowtie1 with parameters “ -m 1–best–strata -S–chunkmbs 200”^72^. MACS2 was used to call peaks compared to input using *q*_value_thresholds = 0.05, 0.01, 0.001. The aligned reads with at least two biological replicates were processed using the irreproducibility discovery rate (IDR)^73^. The *p*-value cutoff was 1e-16. Peaks were then annotated using ChIPpeakAnno package. In order for us to classify a peak as associating with a gene, the peak summit must have been within 1.5 kb upstream and downstream of the gene’s annotation. The gene length was normalized to 1 kb to describe average enrichment of peaks around gene body TSS. The B-ARR-6-BA motif matrix was used by Homer^74^ to identify the locations of the motif sequence on the whole genome. Promoter regions were defined as −1500 to +100 bps relative to the TSS. Gene ontology analysis was conducted using DAVID GO^37;^ the top 3000 genes ranked by IDR peak score of each experiment were evaluated for GO term overrepresentation (The *p*-values were corrected for multiple testing). Total 200 bps flanking the binding summits of 1000 top-ranking peaks for each experiment were also used for identification of each DNA binding motif using MEME-ChIP suite^75^. The list of genes associated with cytokinin pathway was downloaded from Gene Ontology Consortium (http://www.geneontology.org)^76^. The heatmap of the B-ARRs’ target genes was hierarchical clustered based on the Euclidean distances, calculated from the number of shared targets between each factor for different conditions. A directed network was constructed based on the relationship between TFs and their binding targets by igraph (http://igraph.org)^77^ with nodes representing either *type-A ARR* or TFs with significant transcriptional changes defined by the meta-analysis (Supplementary Data 5) and edges representing either ChIP-Seq binding of type-B ARR or DAP-Seq^33^ binding of TFs. The network was visualized in Cytoscape (v3.4.0)^78^. DAP-Seq data were downloaded from the website (http://neomorph.salk.edu/dap_web/pages/index.php) and only samples without “amp” label were used for this analysis^33^.

### RNA-seq and data analysis

To compare the *arr1/10/12* triple mutant with the wild type, three-days-old seedlings were collected without any treatment. For cytokinin treatment, 10 µM 6-BA or an equal volume of ethanol was applied to each sample. RNA was isolated using RNeasy plant kit (Qiagen, cat #74904) and libraries were prepared by NeoPre Library Prep System (Illumina). Alignments were done by Tophat 2(v2.0.8, using TAIR10, Bowtie 2, and default parameters)^79^ and differential expression was called by CuffDiff (Cufflinks v2.1.1, using TAIR10 and default parameters)^80^. The significantly differentially expressed genes used 1.6-fold change and *q*-value <= 0.05 as cutoff.

### Data availability

Raw and processed data can be found with GEO deposition accession number GSE94486. The authors declare that all other data supporting the findings of this study are available within the manuscript and its supplementary files or are available from the corresponding author upon request.

### Electronic supplementary material

Supplementary Information

Description of Additional Supplementary Files

Supplementary Data 1

Supplementary Data 2

Supplementary Data 3

Supplementary Data 4

Supplementary Data 5

Supplementary Data 6

Supplementary Data 7

Supplementary Data 8

Supplementary Data 9

Supplementary Data 10
