# Author Correction: A B-ARR-mediated cytokinin transcriptional network directs hormone cross-regulation and shoot development

**DOI:** 10.1038/s41467-023-41571-5

**Published:** 2023-09-26

**Authors:** Mingtang Xie, Hongyu Chen, Ling Huang, Ryan C. O’Neil, Maxim N. Shokhirev, Joseph R. Ecker

**Affiliations:** 1 Plant Biology Laboratory, and Genomic Analysis Laboratory, The Salk Institute for Biological Studies, La Jolla, CA 92037 USA; 2 Howard Hughes Medical Institute, The Salk Institute for Biological Studies, La Jolla, CA 92037 USA; 3 Department of Computer Science, Dartmouth College, Hanover, NH 03755 USA; 4 The Razavi Newman Integrative Genomics and Bioinformatics Core Facility, The Salk Institute for Biological Studies, La Jolla, CA 92037 USA; 5 Bioinformatics Program, University of California at San Diego, La Jolla, CA 92093 USA

Correction to: *Nature Communications* 10.1038/s41467-018-03921-6, published online 23 April 2018

The original version of this Article contained two errors in Fig. 1c. In the top row of the second panel, the image of ARR1+BA (root hair) was inadvertently replicated, using the image that appears in the bottom panel depicting ARR12+BA (root hair). Similarly, in the third panel, the top row featuring ARR1+BA (hypocotyl) was mistakenly duplicated with an image sourced from the bottom row of the same panel, which depicts ARR12+BA (hypocotyl). These images have been replaced with the correct images in both cases. This error has been corrected in both the PDF and HTML versions of the Article.

