# Supplementary material for: A B-ARR-mediated cytokinin transcriptional network directs hormone cross-regulation and shoot development
Source: Nat Commun. 2018 Apr 23;9:1604. doi: 10.1038/s41467-018-03921-6 (PMC5913131; doi:10.1038/s41467-018-03921-6)
Supplement: Supplementary file 1 — Supplementary Information [file 41467_2018_3921_MOESM1_ESM.pdf]

Supplementary Figures

Supplementary Fig. 1

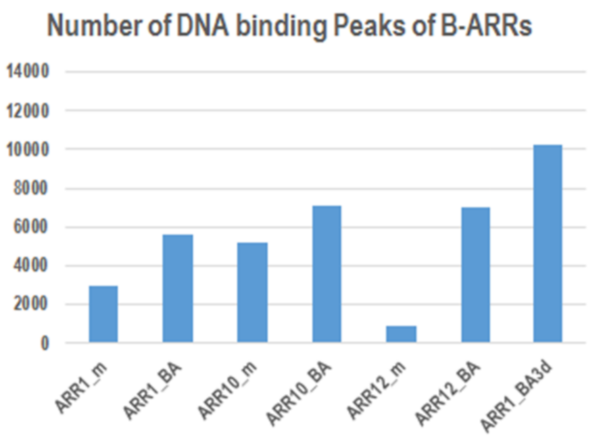

Supplementary Fig. 1 B-ARRs DNA binding peaks The number of conservative (narrow) binding sites bound by each B-ARR at endogenous level of cytokinin (m), treated with 10 µM 6-BA for 4 hours (BA), and treated with 10 µM 6-BA for 3 days (BA3d) under long day (16-hour light / 8-hour dark cycle) condition.

Supplementary Fig. 2

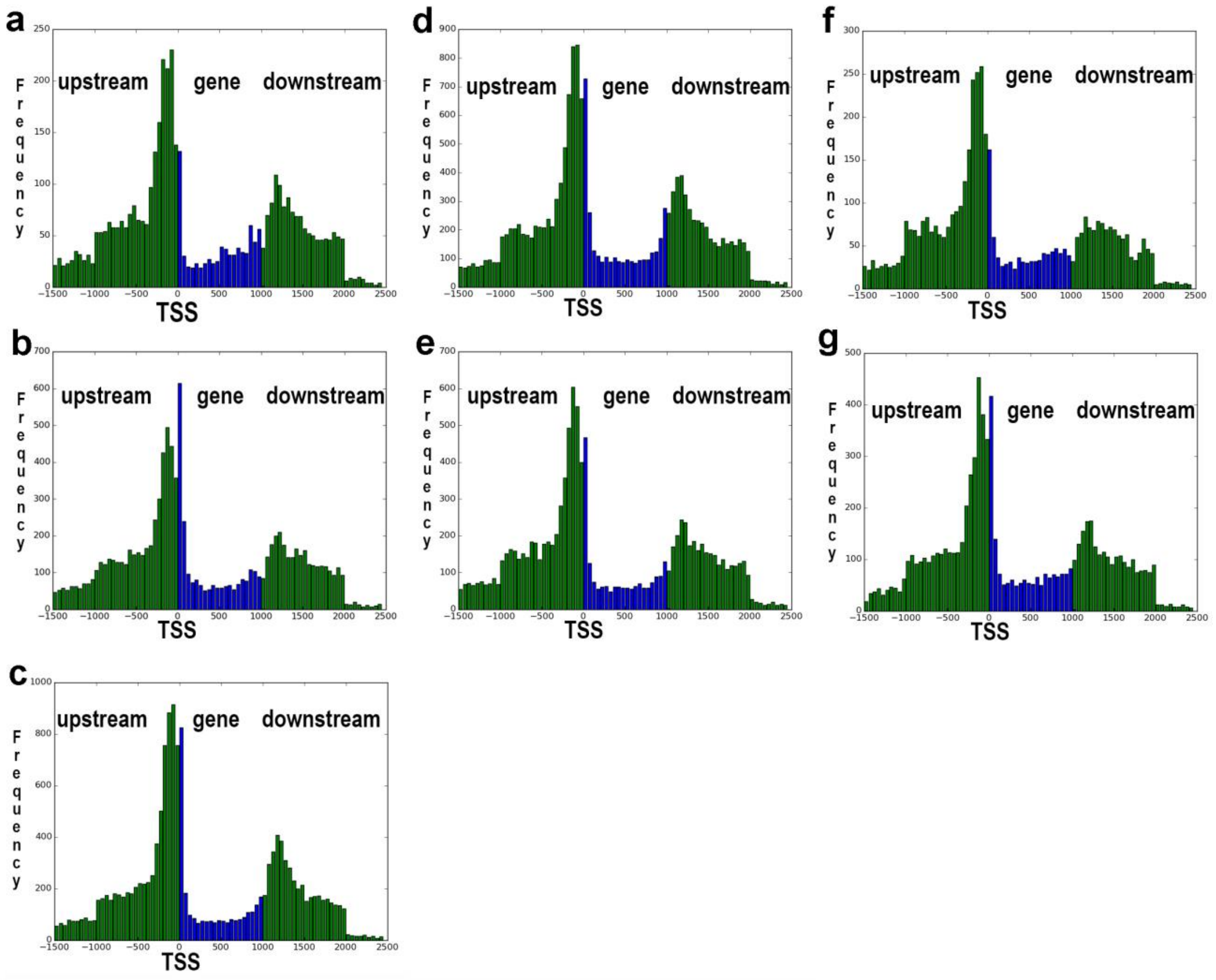

Supplementary Fig. 2 Enrichment around TSS of ChIP-seq peaks The x-axis positions indicate the base distances to the TSS (transcription start site) (TSS at 0). (a) ARR1_m. (b) ARR1_BA. (c) ARR1_BA3d. (d) ARR10_m. (e) ARR10_BA. (f) ARR12_m. (g) ARR12_BA. (mock treatment [m], treatment with 10 µM 6-BA for 4 hours [BA], or treatment for 3 days [BA3d]).

Supplementary Fig. 3

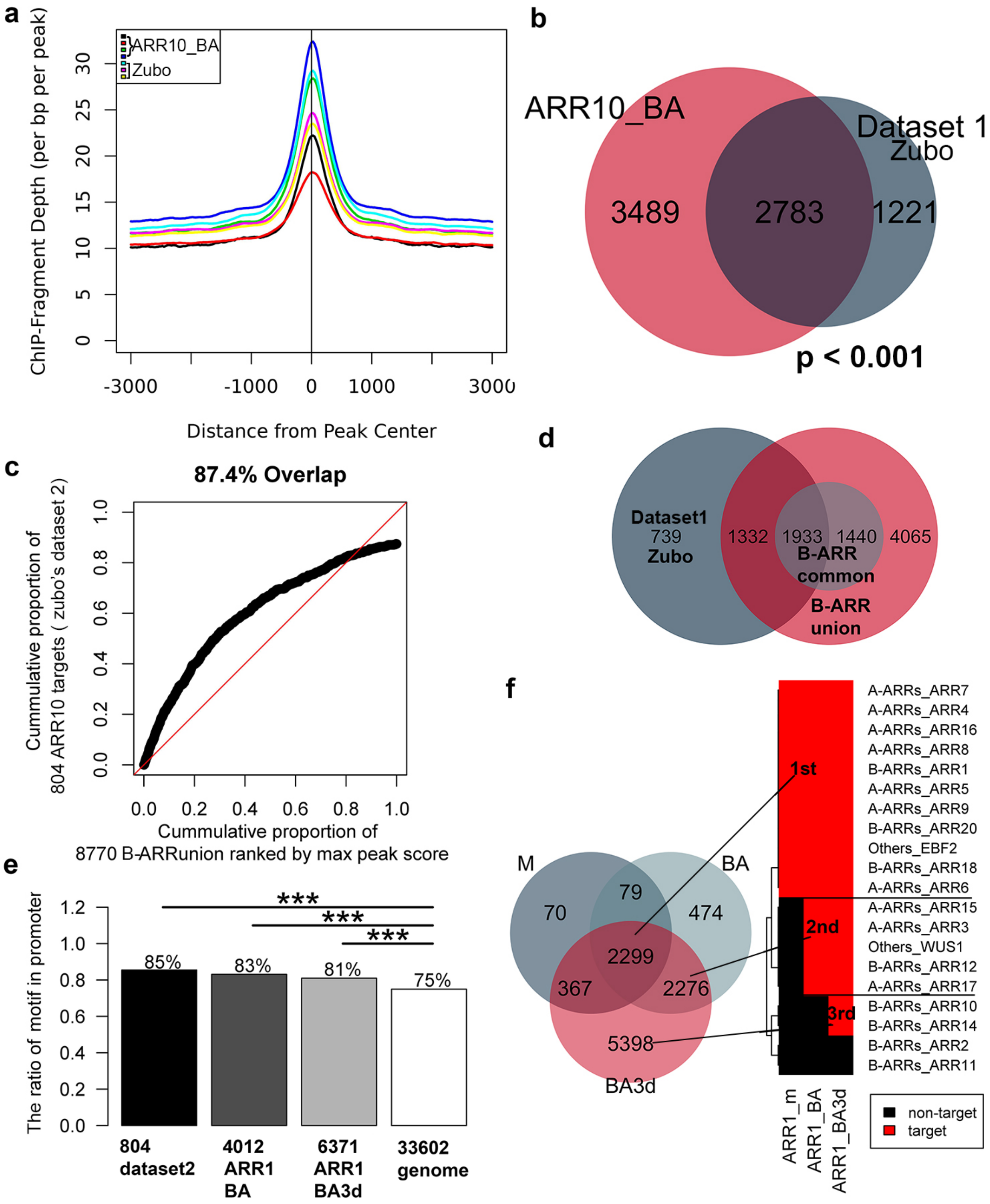

Supplementary Fig. 3 Comparison between Zubo datasets and results in this study (a) Overall read density in the peak regions centered at the ARR10_BA (ARR10 with 4-hour 6-BA treatment) peaks, each color represented one biological replicate. (b) Venn diagram showing significant overlap between Zubo's potential targets dataset 1 (ARR10 over-expression line) with

the candidate targets of ARR10_BA in this study (Fisher's Exact Test, p-value < 0.001). (c) A cumulative proportion plot showing 87.4% overlap between Zubo's dataset 2 (ARR10 over-expressing targets with transcriptional changes) and 8770 B-ARR union targets in this study, showing enriched overlapping B-ARR binding signals in gene targets with higher peak scores (steeper slope at the left). (d) Venn diagram showing relationship between Zubo's dataset 1 and B-ARR common and union targets. (e) The proportion of B-ARR-6-BA motif at promoters (-1.5 kb to +100 bp) of ARR1_BA and ARR1_BA3d targets compared to Zubo's ARR10 "true targets" (dataset 2) and Arabidopsis genome background (*** indicating Fisher's Exact Test, p < 0.001). (f) A Venn diagram showing the overlap of ARR1 binding to their targets at endogenous level (mock (m)), response to short (4 hours(BA)), and longer BA treatment (3 days(BA3d)) and a hierarchical ranking of these bindings.

Supplementary Fig. 4

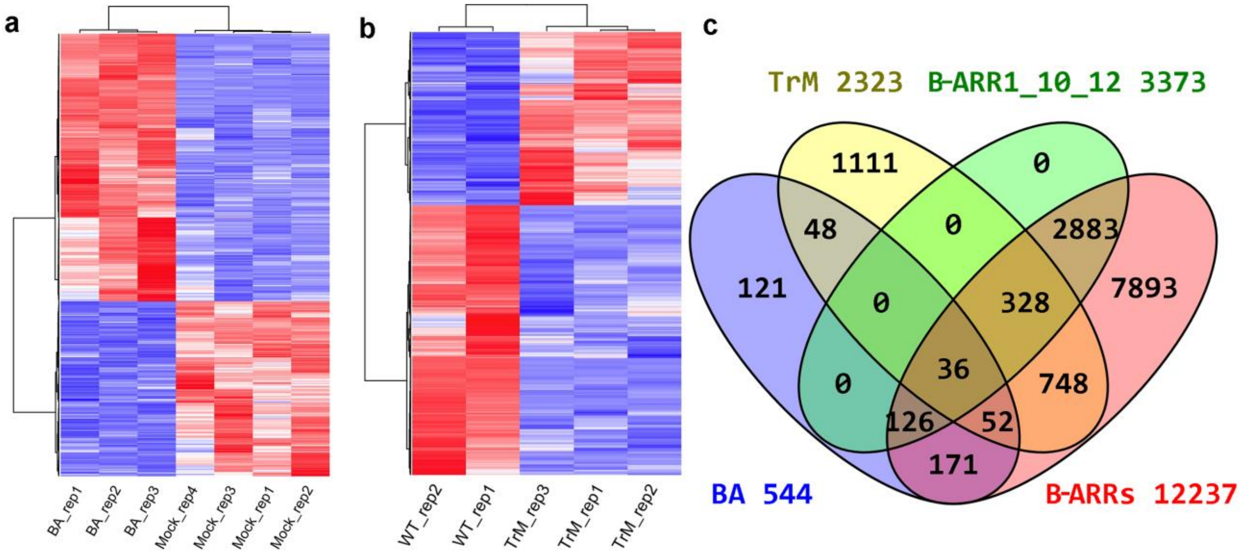

Supplementary Fig. 4 Gene expression by cytokinin treatment and in *arr1/10/12* mutants (a) Heatmap of cytokinin response genes treated with 10 µM 6-BA for four hours. (b) Heatmap of differentially expressed genes in *arr1/10/12* triple mutants. (c) Venn diagram showing B-ARR targets were regulated either by 6-BA treatment (544 genes) or in *arr1/10/12* triple mutants (2323 genes). Candidate B-ARR targets in any condition (12237 genes that did not apply 1.5 kb cutoff). ARR1_10_12 common targets in BA treatment (3373 genes).

Supplementary Fig. 5

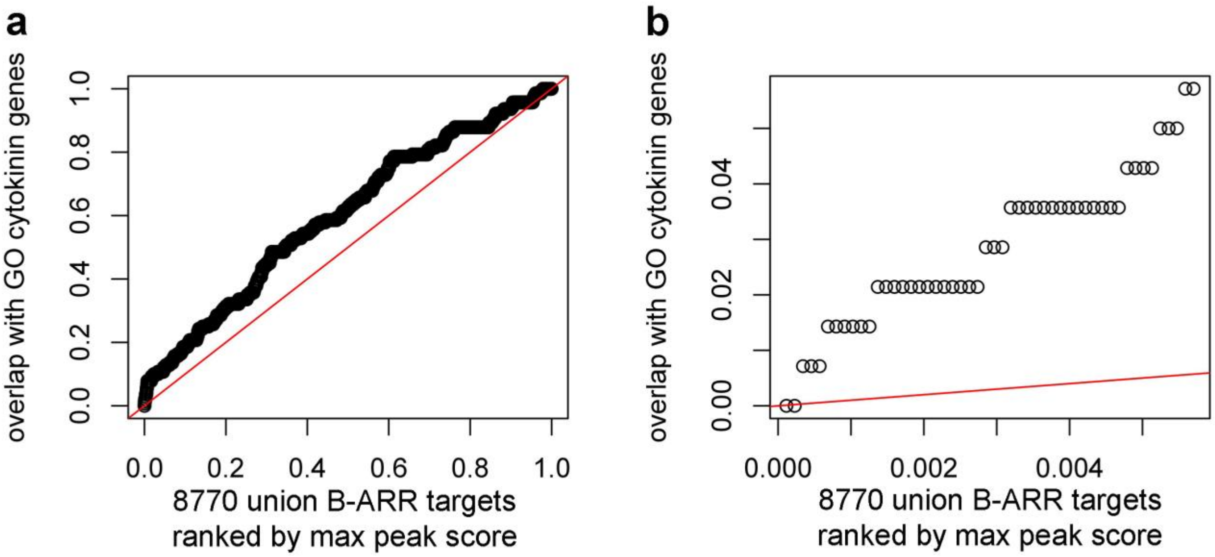

Supplementary Fig. 5 Top-ranking B-ARR targets are related to cytokinin pathways (a) A cumulative plot shows the overlap between GO cytokinin pathway genes and all 8770 union B-ARR targets ranked by the max peak score of three 6-BA treated B-ARRs. (b) Only top 50 genes ranked by the max peak scores in the union B-ARR targets of the cumulative plot. The red line represents the expected overlap with cytokinin pathway genes in the total list assuming random distribution ($p < 0.001$, binomial test).

Supplementary Fig. 6

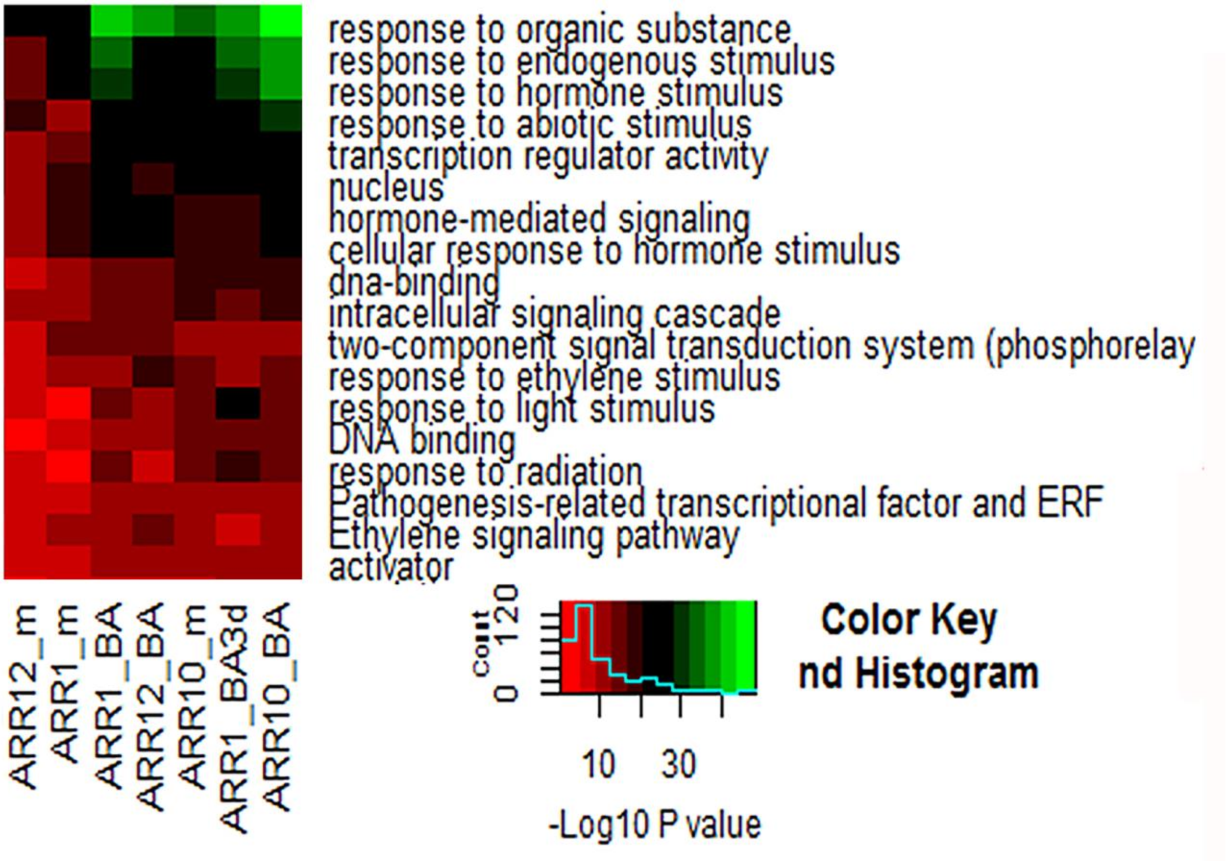

Supplementary Fig. 6 Gene ontology analysis of all B-ARRs The -log10 adj. P value of top 18 GO terms presented as a heatmap. The sample labels were the same as Fig. 2c.

Supplementary Fig. 7

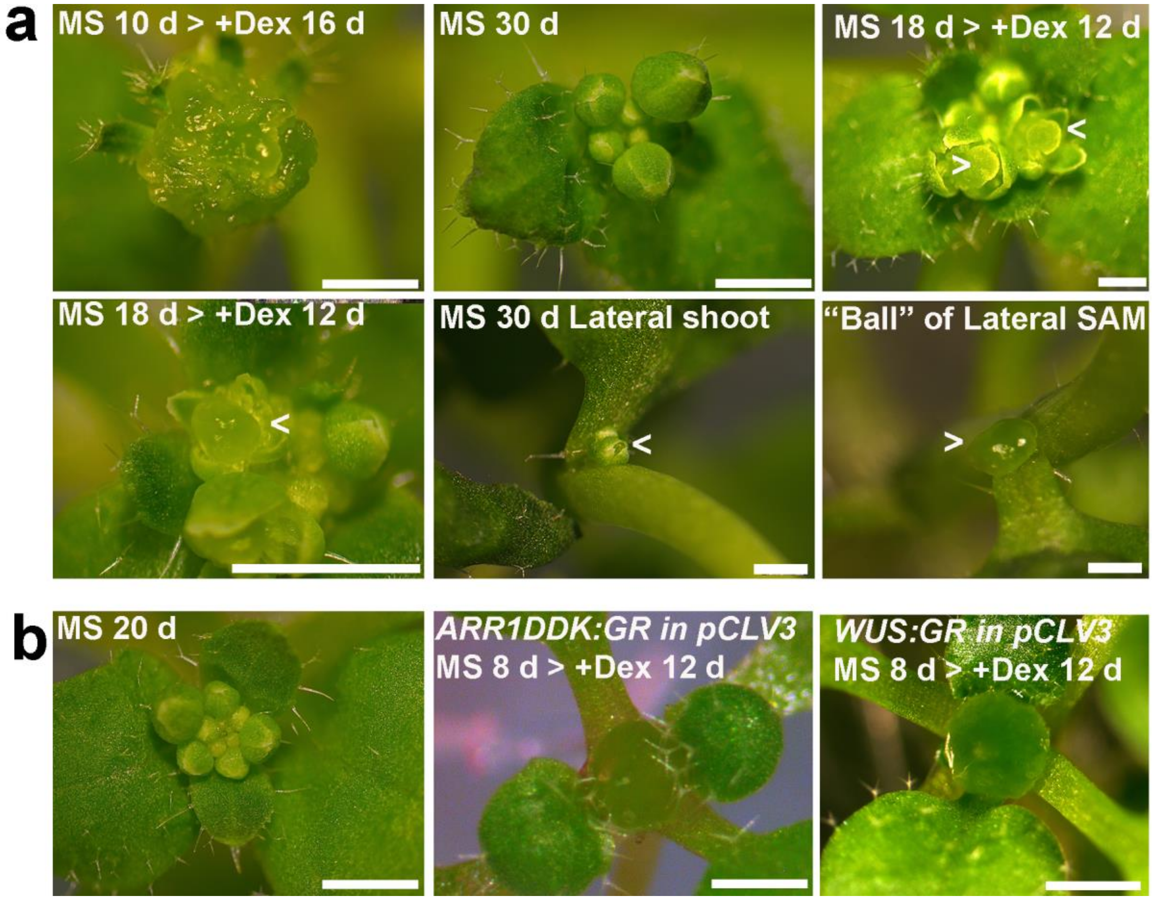

Supplementary Fig. 7 Phenotypes of expressing *ARR1ΔDDK* or *WUS* in stem cells (a) Adult plant phenotypes of the expression of *ARR1ΔDDK in pCLV3* domain (The same line used in Fig. 6e). Seeds were germinated on MS plates for 10 days then transferred to 10 µM Dex plates for 16 days (MS 10 d > +Dex 16 d). Seedlings were grown on MS for 18 days until bolting then transferred to 10 µM Dex plate for 12 days, showing indeterminate floral meristem (MS 18 d > +Dex 12 d, arrow), “ball” of the lateral shoot apical meristem (SAM) (“Ball” of Lateral SAM), and 30-day uninduced control plants (MS 30 d and MS 30 d Lateral shoot). (b) The similarity between *ARR1ΔDDK:GR in pCLV3* (Another independent line) and *WUS:GR in pCLV3*. Seeds were germinated on MS for 8 days and transferred to MS for additional 12 days (MS 20 d) or transferred to 10 µM Dex plate for 12 days (MS 8 d > +Dex 12 d). Scale bar = 1 mm.

Supplementary Fig. 8

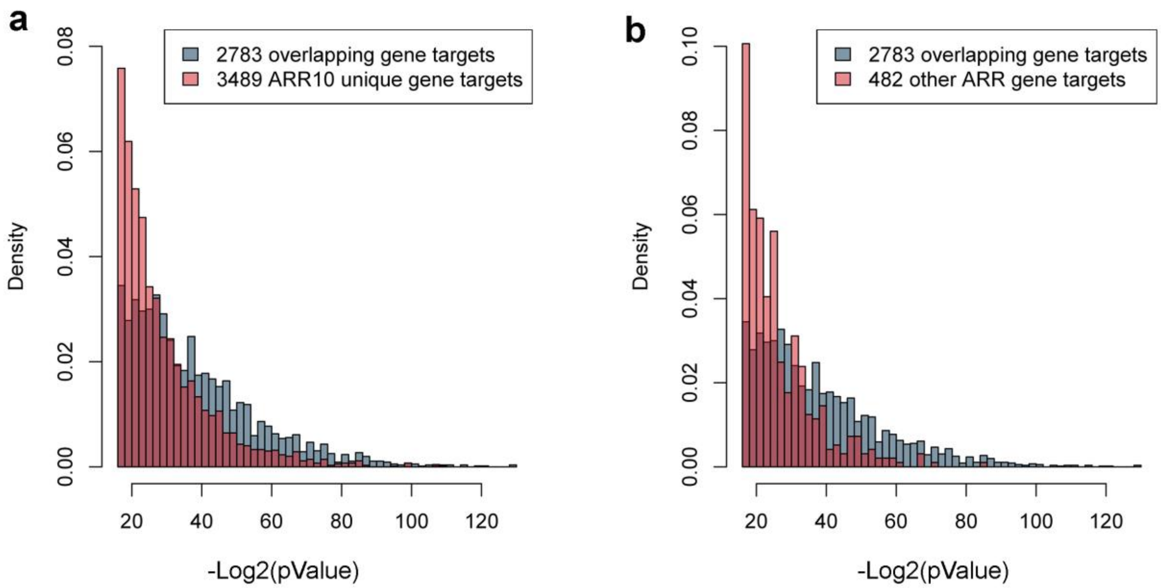

Supplementary Fig. 8 2783 overlapping target genes have higher peak scores (a) Histogram of peak scores distribution of 3489 unique ARR10_BA target genes that were not identified by ARR10 over-expressing dataset and 2783 overlapping target genes shared with ARR10 over-expressing dataset. (b) Histogram of peak score distribution between 482 ARR10 over-expressing target genes that were identified by ARR1_BA or ARR12_BA and 2783 overlapping target genes. -Log10(pValue) = peak scores.
