# Supplementary material for: A B-ARR-mediated cytokinin transcriptional network directs hormone cross-regulation and shoot development
Source: Nat Commun. 2018 Apr 23;9:1604. doi: 10.1038/s41467-018-03921-6 (PMC5913131; doi:10.1038/s41467-018-03921-6)
Supplement: Supplementary file 2 — Description of Additional Supplementary Files [file 41467_2018_3921_MOESM2_ESM.pdf]

Description of Additional Supplementary Files

Supplementary Data 1

Candidate targets of B-ARRs

Supplementary Data 2

3373 ARR1_ARR10_ARR12 common targets

Supplementary Data 3

Differentially expressed genes with cytokinin treatment

Supplementary Data 4

Differentially expressed genes in *arr1/10/12* triple mutants

Supplementary Data 5

Core B-ARR target genes compared with transcriptomic studies

Supplementary Data 6

A graph of the B-ARRs directed gene regulatory network

Supplementary Data 7

Plant hormone pathway genes targeted by B-ARR_1_10_12

Supplementary Data 8

Ratio of ARR1 binding to upstream and downstream of targets

Supplementary Data 9

B-ARR binding locations to B-ARR targets

Supplementary Data 10

Overlap between ARR10 over-expression and ARR10_BA targets
